# A gene network regulating echinocandin heteroresistance in *Candida glabrata*

**DOI:** 10.1128/aac.00549-26

**Published:** 2026-10-08

**Authors:** Abigail A. Harrington, Kyle W. Cunningham

**Affiliations:** 1 Department of Biology, Johns Hopkins University 1466, Baltimore, Maryland, USA; University of Iowa, Iowa City, Iowa, USA

**Keywords:** regulatory mechanisms, *Candida*, heteroresistance, echinocandins

## Abstract

While pathogenic fungi can acquire resistance to the current arsenal of antifungals through genetic mutations, heteroresistance has emerged as an important new cause of therapeutic failures. Heteroresistance is generally thought to arise in small subpopulations that display phenotypic resistance to antifungals without genetic mutations. This study blurs that line by showing gain-of-resistance (GOR) mutations in *FKS2*, which encodes a target of echinocandins and fungerps, causing broad heteroresistance in *Candida glabrata*. Identical gain-of-resistance mutations in *FKS1*, a paralog of *FKS2*, caused resistance and little or no heteroresistance. Heteroresistance of *FKS2-GOR* strains decreased when stress-responsive transcription factors (Crz1 and Rlm1) known to increase *FKS2* expression were eliminated and was nearly abolished when the upstream regulators (calcineurin and Slt2) were mutated or inhibited. Conversely, stresses and mutations known to increase *FKS2* expression also increased the survival of *FKS2-GOR* mutants exposed to high doses of echinocandins. A genome-wide screen using transposon sequencing revealed additional regulators of heteroresistance and resistance. Mutants lacking *DFG5* or *SSK2* exhibited strongly increased *FKS2-GOR* function without increased expression. Mutants lacking *IRA1*, an inhibitor of the Ras1-PKA signaling pathway that senses glucose availability, decreased expression of *FKS2* and increased expression of *FKS1*, thereby decreasing heteroresistance and increasing resistance, respectively, when these genes carried GOR mutations. The findings suggest that heterogeneous expression of resistance genes and heterogeneous regulation of their products combine to produce echinocandin heteroresistance. Analogous mechanisms may facilitate heteroresistance to azoles and echinocandins in other *Candida* species.

## INTRODUCTION

*Candida glabrata* (a.k.a. *Nakaseomyces glabratus*) is an opportunistic fungal pathogen of increasing clinical concern. *C. glabrata* is the second most common causative agent of candidiasis, behind *Candida albicans*, which has led the World Health Organization to list *C. glabrata* as a high-priority threat. Recent surveillance in the United States indicated that *C. glabrata* was the most common cause of candidemia-related hospitalizations in 2024 (1). This is in part due to its innate resistance to azoles and rapid acquisition of resistance to echinocandins (2–4), which results in high rates of therapeutic failure (5, 6). Resistance refers to the ability of cells to grow in higher doses of drug and is typically the result of genetic gain-of-resistance (GOR) mutations in the drug target, or mutations in other components that increase drug efflux, prevent drug influx, or otherwise sequester the drug such that the typical drug dose is no longer adequate for complete clearance. Echinocandins and fungerps target essential enzymes in the plasma membranes of fungal cells that are responsible for the synthesis of beta-1,3-glucan. In *C. glabrata*, the catalytic subunits of the synthases are encoded by two functionally redundant genes (*FKS1* and *FKS2*) that originated from an ancient whole-genome duplication event (7), with a third homologous gene (*FKS3*) playing no detectable role (8). *FKS1* expression is thought to be constitutive and unresponsive to cell wall stresses, while *FKS2* expression is highly responsive to stresses, particularly through the calcineurin and cell wall integrity (CWI) signaling pathways (8–10). Strong resistance to echinocandins usually arises through non-synonymous mutations in either *FKS1* or *FKS2* within two different “hotspot” regions (6, 11). Many of these hotspot mutations confer cross-resistance to multiple echinocandins (caspofungin, micafungin, and anidulafungin) and fungerps (enfumafungin and ibrexafungerp) (11–13). Recently, enfumafungin was found to bind directly in the hotspot-1 region of Fks1 and Fks2 enzymes from the model yeast, *Saccharomyces cerevisiae*, with critical interactions between the antifungal and the conserved amino acids F639 and F658, respectively (14). Substitution or deletion of this critical amino acid conferred cross-resistance to all echinocandins in addition to the fungerps without strongly altering enzymatic activity *in vitro* (14). Mutations at the same sites in *FKS1* and *FKS2* genes of *C. glabrata* (F625 and F659) confer similar cross-resistance and arise frequently in clinical isolates from treated patients (8, 15–17).

Heteroresistance refers to a phenomenon in which only a small subpopulation of cells within a clonal population transiently exhibits phenotypic resistance to antifungals in the absence of stable *de novo* mutations in the genome. Heteroresistance differs from persistence, in which a small subpopulation survives for a longer period of time in the presence of excess antifungal than the main population in time-kill assays (18, 19). Though heteroresistance has been associated with therapeutic failures, the epigenetic mechanisms that govern the phenotype remain poorly defined in pathogenic yeasts. Heteroresistance to azole-class antifungals such as fluconazole has been observed frequently in clinical isolates of *Cryptococcus* and attributed to rare duplications of a whole chromosome expressing the drug target and a drug efflux gene (20–23). Heteroresistance to fluconazole in *C. glabrata* was associated with increased drug efflux and difficult-to-treat infections *in vivo* (18). However, heteroresistance to echinocandins was not detected in several isolates of *C. glabrata* (18, 19) and has not yet been reported in *C. albicans* (24). Importantly, several clinical isolates of *Candida parapsilosis* and *Candida auris* were recently shown to exhibit clear heteroresistance to echinocandins leading to breakthrough infections (25).

Resistance to echinocandins can be quantified accurately using growth assays, such as broth microdilution assays, E-tests, and halo assays, with the relevant parameter for comparison being the minimum inhibitory concentration (MIC) or the drug concentration causing 50% growth inhibition (IC50). Though growth assays can sometimes provide information on minor subpopulations, heteroresistance is quantified most accurately and directly by the population analysis profile (PAP) assay (26–28). This method relies on serially diluting clonal populations of fungal cells, plating on agar media containing serial dilutions of antifungal drugs, counting visible colonies after an appropriate incubation period, and calculating the percentage of colony-forming units (CFUs) relative to the untreated population. Non-heteroresistant isolates exhibit nearly all-or-none behavior at transitional doses, with colony-forming units (CFUs) near 100% or 0% relative to the untreated population. Heteroresistant isolates exhibit CFUs between 50% and 0.0015% (limit of detection) over a broad range of doses. In the case of *C. parapsilosis* exposed to echinocandins, some isolates exhibited a gradual decline in CFUs within the transitional range (TR) of doses, while others exhibited step-like declines (25), suggesting complexity in the underlying regulatory mechanisms.

This study shows that *C. glabrata* strains with gain-of-resistance (GOR) mutations in *FKS2*, but not *FKS1*, exhibit strong heteroresistance to echinocandins. The gradual decline of CFUs over the transitional range of doses suggested that the cause of progressive heteroresistance may be variable expression and/or function of the *FKS2-GOR* gene product in the clonal populations. This hypothesis was supported genetically by introducing mutations in the calcineurin-Crz1 and Slt2-Rlm1 signaling pathways shown previously to increase *FKS2* expression (8). Chemical stresses that increase *FKS2-GOR* expression exacerbated heteroresistance, while chemically decreasing *FKS2* expression attenuated heteroresistance to the echinocandin micafungin. A genome-wide transposon sequencing (Tn-seq) screen in the heteroresistant strain *FKS2-F659Δ* was performed to identify additional regulators of heteroresistance. *IRA1*, predicted to encode an inhibitor of the small GTPase Ras1 (29), was found to promote heteroresistance in *FKS2-F659Δ* but decrease resistance in *FKS1-F625Δ* likely through effects on *FKS* gene expression. *SSK2* and *DFG5* appeared to antagonize *FKS2-GOR* function at post-transcriptional levels. These findings begin to unravel mechanisms of echinocandin heteroresistance in pathogenic yeasts, which could potentially be targeted by the calcineurin inhibitor FK506 or other small molecules to help manage recalcitrant infections.

## RESULTS

### Gain-of-resistance mutations in *FKS2* confer progressive heteroresistance to echinocandins

**Fig 1:**
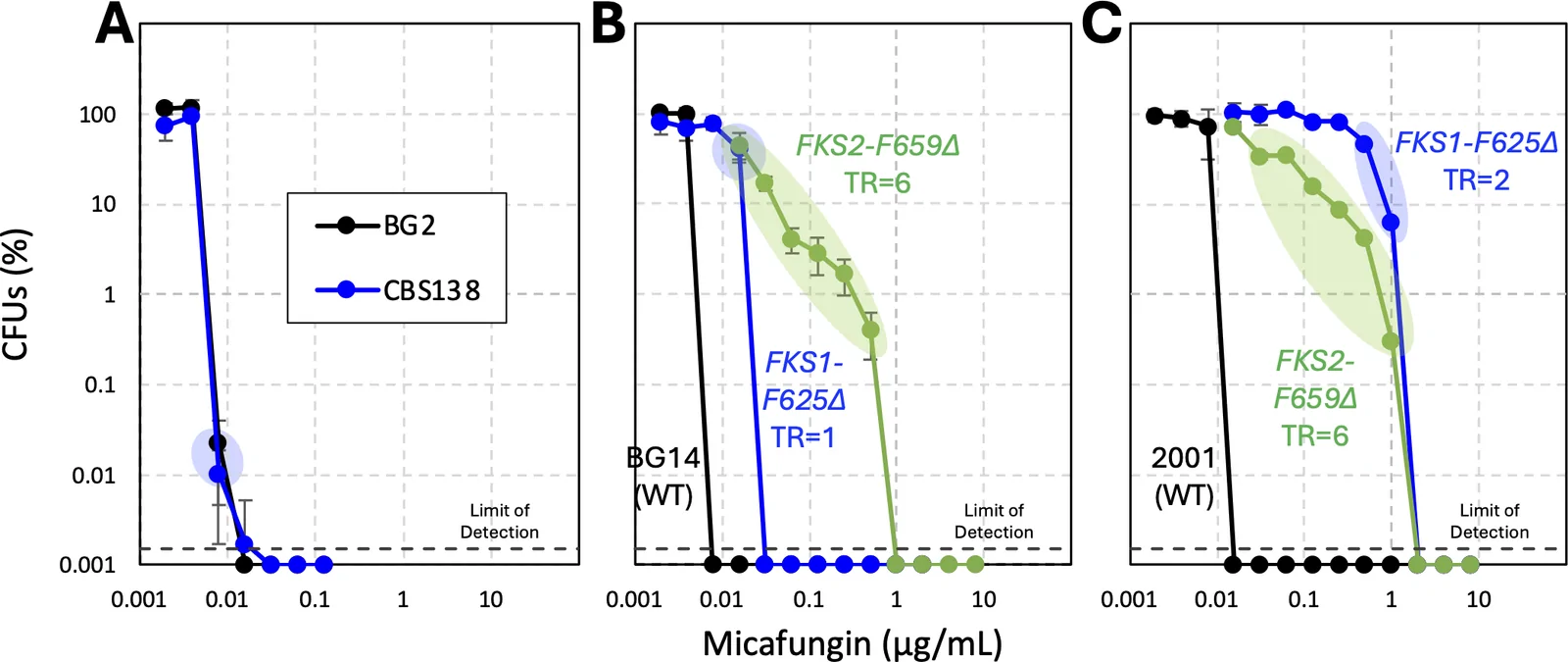
*C. glabrata* strains bearing *FKS2-F659∆,* but not *FKS1-F625∆,* exhibited strong progressive heteroresistance to micafungin. Wild-type clinical isolates (**A**) and derivatives carrying the same GOR mutation in *FKS1* and *FKS2* (**B and C**) were analyzed using the PAP assay. Replicate colonies were grown to saturation in SCD medium, serially diluted, and then plated on YPD agar media containing varying doses of micafungin. After 24 h of incubation at 30°C, colonies were counted using a dissecting microscope. The percent CFUs for each strain at every dose were calculated with respect to the no-drug control. The average CFUs of four biological replicates (±SD) is shown. Limit of detection = 0.0015%, or one colony detected out of four undiluted biological replicates.

Numerous clinical isolates of *C. glabrata* have been reported as heteroresistant to fluconazole (18), but heteroresistance to echinocandins has not been reported. Two commonly used clinical isolates were tested in population analysis profile (PAP) assays for heteroresistance: BG2, a vaginal isolate, and CBS138, a gastrointestinal isolate. As expected, both strains exhibited no heteroresistance to micafungin (Fig. 1A). Loss-of-function mutations in *MRP20*, *ALG6*, and *PDR1* were previously shown to mildly alter resistance to echinocandins in the BG2 strain background (30). Deletion mutants lacking these genes, as well as a *PDR1-GOF* (gain-of-function) mutant that increased echinocandin resistance (30), did not exhibit altered heteroresistance to micafungin (Fig. S1). These findings show that echinocandin resistance does not always confer heteroresistance in *C. glabrata*.

Many isolates of *C. parapsilosis* exhibited heteroresistance to echinocandins (25). As all isolates of this species bear a natural cross-resistance mutation in hotspot-1 of its *FKS1* gene (31), we tested whether hotspot-1 mutations in *FKS1* and *FKS2* genes of *C. glabrata* can confer heteroresistance in addition to resistance. Strikingly, the *FKS2-F659∆* mutant derivative from strain BG2 exhibited broad heteroresistance to micafungin, whereas deletion of the same codon in *FKS1* (*FKS1-F625∆*) exhibited only slight heteroresistance (Fig. 1B). The transitional range (TR) where CFUs varied from 50% maximum to the limit of detection was quantified by counting the doses as previously described (32). Note that 2^TR^ equals the fold-change in antifungal doses where heteroresistance is observed. The *FKS2-F659∆* mutant exhibited TR = 6, while the *FKS1-F625∆* mutant exhibited TR = 1, in comparison to the parent strain where TR = 1. Similar results were found with the same mutants in the 2001-HTU strain background (Fig. 1C), a well-studied derivative of CBS138 (33–35). Similar results were also obtained using caspofungin instead of micafungin in the agar medium (Fig. S2). Additionally, heteroresistance to micafungin was observed when several other gain-of-resistance substitution mutations were introduced into hotspot-1 of *FKS2*, but not when introduced into *FKS1*, albeit over a narrower TR (Fig. S3). Though these hotspot-1 mutations conferred elevated resistance to echinocandins in broth microdilution assays (Fig. S4A) as described previously (11, 12, 36–38), only the *FKS2-GOR* mutants exhibited broad heteroresistance. The broadest TR was associated with *FKS2-F659∆* mutants, in which the key amino acid residue required for binding of fungerps and echinocandins has been eliminated (8, 14–16). The gradual decline of CFUs observed over six doses of micafungin is reminiscent of progressive heteroresistance observed in some isolates of bacteria exposed to antibiotics (39). Progressive heteroresistance typically involves variable expression of resistance genes in clonal populations, thus producing a broad spectrum of resistance states that are unstable and easily reversible upon passaging in drug-free culture medium (39).

**Fig 2:**
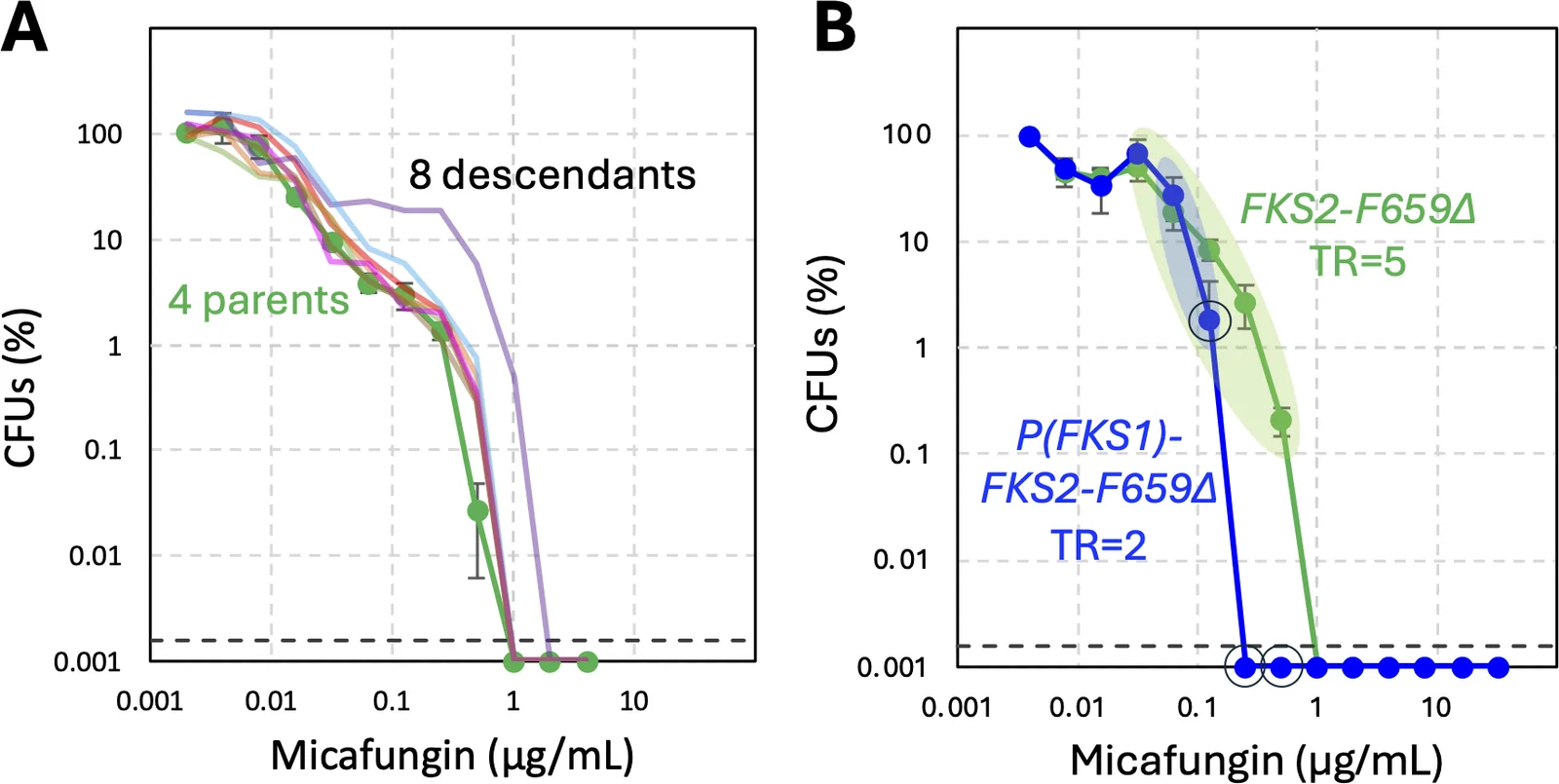
Echinocandin heteroresistance is unstable and partially controlled by the promoter of *FKS2.* (**A**) PAP assays were performed on four replicate colonies of *FKS2-F659∆* mutants and averaged (±SD) (green symbols) as well as eight descendant colonies (individual lines) that had been picked from the PAP assay plate containing 0.25 µg/mL micafungin and passaged in drug-free medium. (**B**) PAP assays were performed on four replicate cultures of *FKS2-F659∆* mutants (green symbols) and promoter-swapped *P(FKS1)-FKS2-F659∆* mutants (blue symbols). The percent CFUs were calculated with respect to 0.003 µg/mL micafungin, and the average CFU ± SD was charted. Open circles indicate samples in the transitional range where the *P(FKS1)-FKS2-F659∆* strain differed significantly from the control strain (*P* < 0.05; Welch’s *t*-test).

To test whether the echinocandin heteroresistance in *C. glabrata* is reversible, eight individual colonies of the *FKS2-F659∆* mutant were picked from agar plates containing 0.25 µg/mL micafungin, passaged in drug-free culture media, and retested in PAP assays. All eight descendant colonies produced heteroresistance patterns similar to the parent *FKS2-F659∆* strain, though a rightward shift was detected in one descendant (Fig. 2A). All the descendant colonies exhibited similar levels of resistance to micafungin as the parent strains (Fig. S4B). Thus, most colonies that formed in high concentrations of micafungin on PAP assay plates did not contain stable resistance mutations and appeared to readily reverse their phenotypic resistance upon a single passage in drug-free medium.

To test whether the heteroresistant phenotype of *FKS2-F659∆* depends on heterogeneous expression, its promoter sequences were removed and replaced with a 1 kb segment from the constitutive promoter of *FKS1* and introduced the resulting “promoter-swap” plasmid into an *fks2∆* mutant derivative of BG14. In PAP assays, the promoter-swap *pFKS1-FKS2-F659∆* strain exhibited a much narrower transitional range (TR = 2) relative to the unswapped control strain (Fig. 2B), which was comparable to the extremely mild heteroresistance observed for *FKS1-F625∆* mutants (Fig. 1B). However, resistance to micafungin was similar for the promoter-swapped *pFKS1-FKS2-F659∆* strain and the unswapped *FKS2-F659∆* strain (Fig. S4C). Altogether, the findings suggest that the heteroresistant phenotype of *FKS2-F659∆* mutants of *C. glabrata* was highly unstable and reversible and mediated to some degree by the regulatory sequences upstream of the gene.

### Calcineurin and CWI signaling pathways regulate heteroresistance

**Fig 3:**
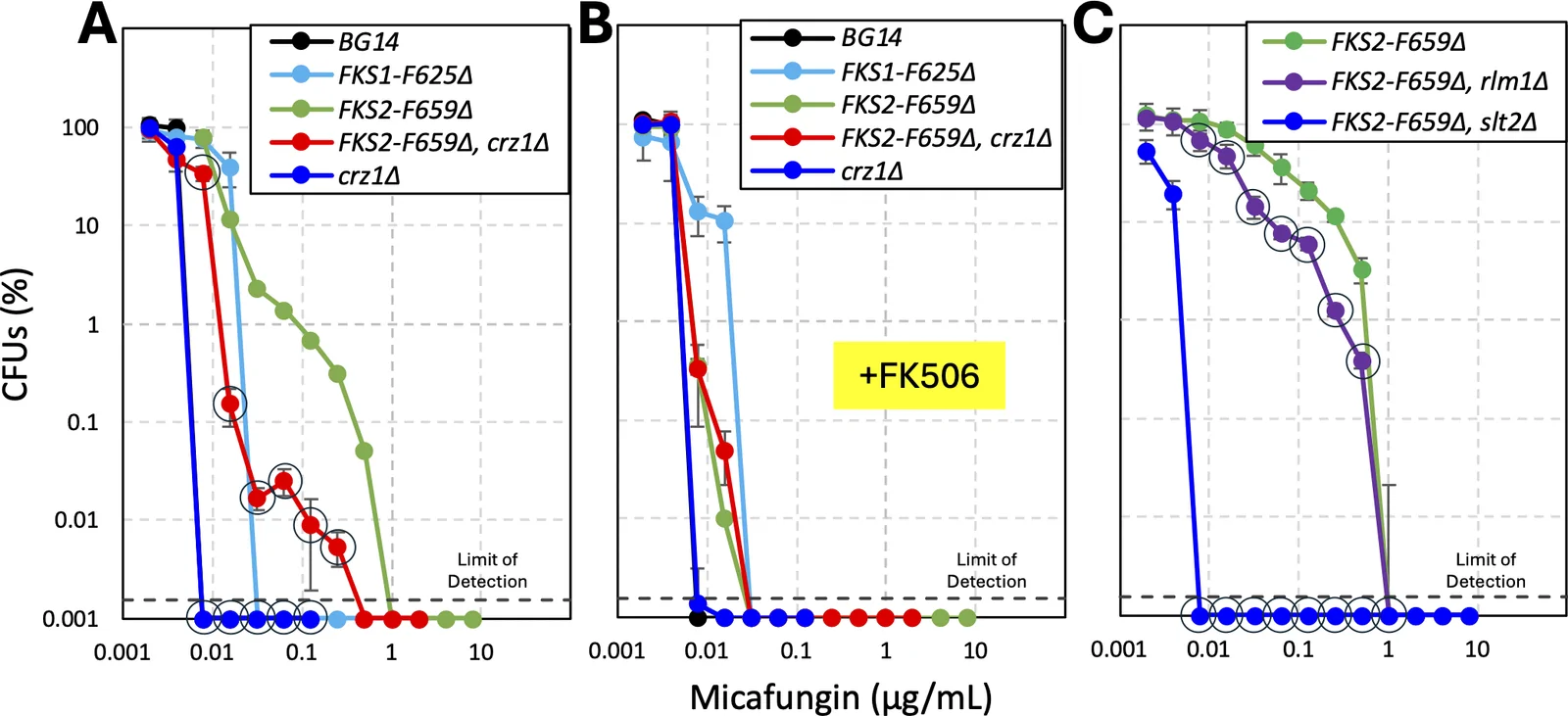
Calcineurin-Crz1 and Slt2-Rlm1 pathways promote heteroresistance in *FKS2-F659∆* strains**.** (**A and C**) PAP assays were performed on the indicated derivatives of strain BG2 as described in Fig. 1, except in (**B**) where agar media were supplemented with 2 µg/mL FK506. Open circles indicate double mutant samples where CFUs differed significantly from the *FKS2-F659∆* samples (*P* < 0.05; Welch’s *t*-test).

*FKS2* expression has been shown to be highly induced upon exposure to stresses, including echinocandin exposure (40, 41), *FKS1* deficiency (42), and endoplasmic reticulum (ER) stressors (40, 43, 44). *FKS2* expression in these conditions depends on several stress-activated signaling pathways such as the calcineurin signaling pathway (8) and the cell wall integrity (CWI) signaling pathway, at least in *S. cerevisiae* (42). Previous work has shown that *FKS2* expression can be highly variable from cell to cell, unlike *FKS1* expression (10). To investigate the effects of calcineurin signaling on heteroresistance, the calcineurin-dependent transcription factor, Crz1, was deleted in the *FKS2-F659Δ* strain background and tested. Interestingly, the *FKS2-F659Δ crz1Δ* double mutant remained heteroresistant to micafungin (TR = 7), but the number of CFUs decreased an average of 80-fold (±SD) over the six transitional doses of micafungin (between 0.015625 and 0.25 µg/mL) (Fig. 3A). Heteroresistance declined profoundly in strains when the calcineurin inhibitor, FK506 (2 µg/mL), was added to the agar media. However, FK506 had little or no effect on the CFUs observed in the *FKS1-F625Δ* mutant (Fig. 3B). These findings suggest the progressive heteroresistance observed in *FKS2-GOR* strains of *C. glabrata* may be partly driven by stress signaling through calcineurin and Crz1 through regulation of expression of *FKS2*, and possibly other target genes (see below).

The effects of CWI signaling on heteroresistance were also investigated by deleting the genes encoding Slt2, a MAP-kinase, and a downstream transcription factor, Rlm1, in the *FKS2-F659Δ* strain background. The *FKS2-F659Δ rlm1Δ* double mutant maintained broad heteroresistance (TR = 6) while exhibiting a 5.3-fold decrease in CFUs compared to *FKS2-F659Δ* (TR = 4) over the transitional range of micafungin (0.015625 and 0.5 µg/mL), indicating this transcription factor produces milder effects than Crz1 (Fig. 3C). However, *FKS2-F659Δ slt2Δ* double mutants exhibited almost no heteroresistance (TR = 1). The *rlm1Δ* and *slt2Δ* gene deletions were also generated in the wild-type parent strain BG14, a *ura3Δ* derivative of BG2, and the *FKS1-F625Δ* mutant backgrounds and tested for changes in heteroresistance and resistance. As expected, these CWI-deficient mutants were not detectably different from their respective parent strains (Fig. S5). Together, these data suggest that major components of the CWI and calcineurin signaling pathways may be required for heteroresistance conferred by *FKS2-GOR* mutations. The milder roles of Rlm1 and Crz1 transcription factors suggest that other outputs of Slt2 and calcineurin may contribute to variable expression and function of *FKS2-GOR* in unstressed populations.

### Population heterogeneity can arise prior to echinocandin exposure

**Fig 4:**
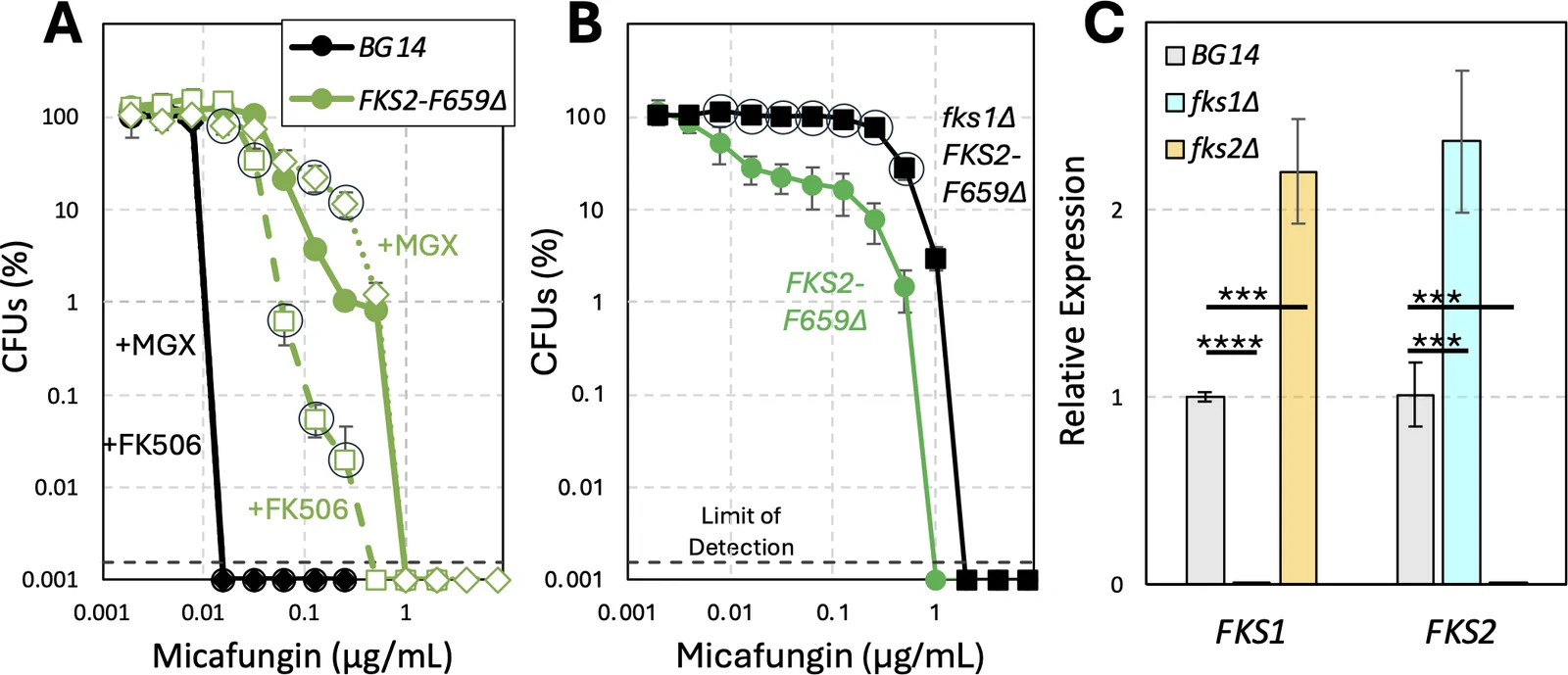
Increased and decreased calcineurin signaling caused increased and decreased heteroresistance, respectively**.** (**A**) Derivatives of strain BG2, BG14, and *FKS2-F659∆* were grown to saturation, then diluted 1:50 into fresh SCD media containing either 1 µg/mL FK506, 0.6 µg/mL manogepix (MGX), or no drug and incubated at 30°C for 2 h. The cells were collected, washed 1× in drug-free fresh medium, resuspended, and tested in PAP assays as described in Fig. 1. (**B**) PAP assays were performed on *FKS2-F659∆* mutants and *fks1∆ FKS2-F659∆* double mutants as described in Fig. 1. Open circles indicate treated or double mutant samples where CFUs differed significantly from control (*P* < 0.01; Welch’s *t*-test). (**C**) qRT-PCR analysis of *FKS1* and *FKS2* expression in the indicated strains (average of 4 replicates, ±SD). ***P < 0.005, ****P < 0.001, respectively; Welch’s *t*-test).

The PAP assay counts colonies that arise from single cells plated on different doses of antifungals. To form a visible colony, the plated cell must survive the antifungal assault and proliferate along with most of its daughter cells. To distinguish whether calcineurin and stress alter heteroresistance of *FKS2-GOR* strains before plating, the *FKS2-F659∆* mutant was exposed for 2 h to either 1 µg/mL FK506 or 0.6 µg/mL manogepix (MGX), an ER stressor that strongly activates calcineurin and increases expression of *FKS2* (40), and then the cells were washed free of drugs and subjected to standard PAP assays. The FK506 pretreatment decreased CFUs 39-fold (averaged between 0.03125 and 0.25 µg/mL, TR = 4), while the MGX pretreatment increased CFUs 5.1-fold (averaged between 0.0625 and 0.5 µg/mL, TR = 4), both relative to the untreated *FKS2-F659Δ* strain (Fig. 4A). FK506 and MGX did not affect heteroresistance of the wild-type parent strain (Fig. 4A). These results indicate that chemically decreasing or increasing calcineurin signaling in the population prior to plating directly influences heteroresistance of *FKS2-F659Δ* mutants.

Several earlier studies have shown that populations of *fks1∆* mutants express much higher levels of *FKS2* mRNA and Fks2 protein largely due to the elevated activation of calcineurin and Crz1 (8–10). Therefore, we expected *fks1∆ FKS2-F659∆* double mutants to produce higher CFU in the transitional range of micafungin than the *FKS2-F659∆* single mutant. PAP assays strongly confirmed this expectation at all doses in the transitional range (Fig. 4B) and further revealed detectable CFUs for the double mutant at an extremely high dose (1 µg/mL) where the single mutant produced no CFUs. This latter effect may be explained by antagonism of drug-free Fks2-F659∆ enzyme activity by drug-bound Fks1 protein, which was reported previously using both broth microdilution assays and *in vitro* enzymatic assays (8, 17). Similar antagonism of Fks1-F625∆ enzyme activity by drug-bound Fks2 has also been observed (8). Quantitative real-time PCR (qRT-PCR) measurements of *FKS1* and *FKS2* gene expression confirmed that loss of one *FKS* gene caused significant increased expression of the remaining *FKS* gene (Fig. 4C). When taken together, the evidence supports a hypothesis where echinocandin heteroresistance observed in *FKS2-GOR* strains is associated with variation in the level of its expression in clonal populations.

### Tn-seq screens reveal additional regulators of echinocandin heteroresistance

**Fig 5:**
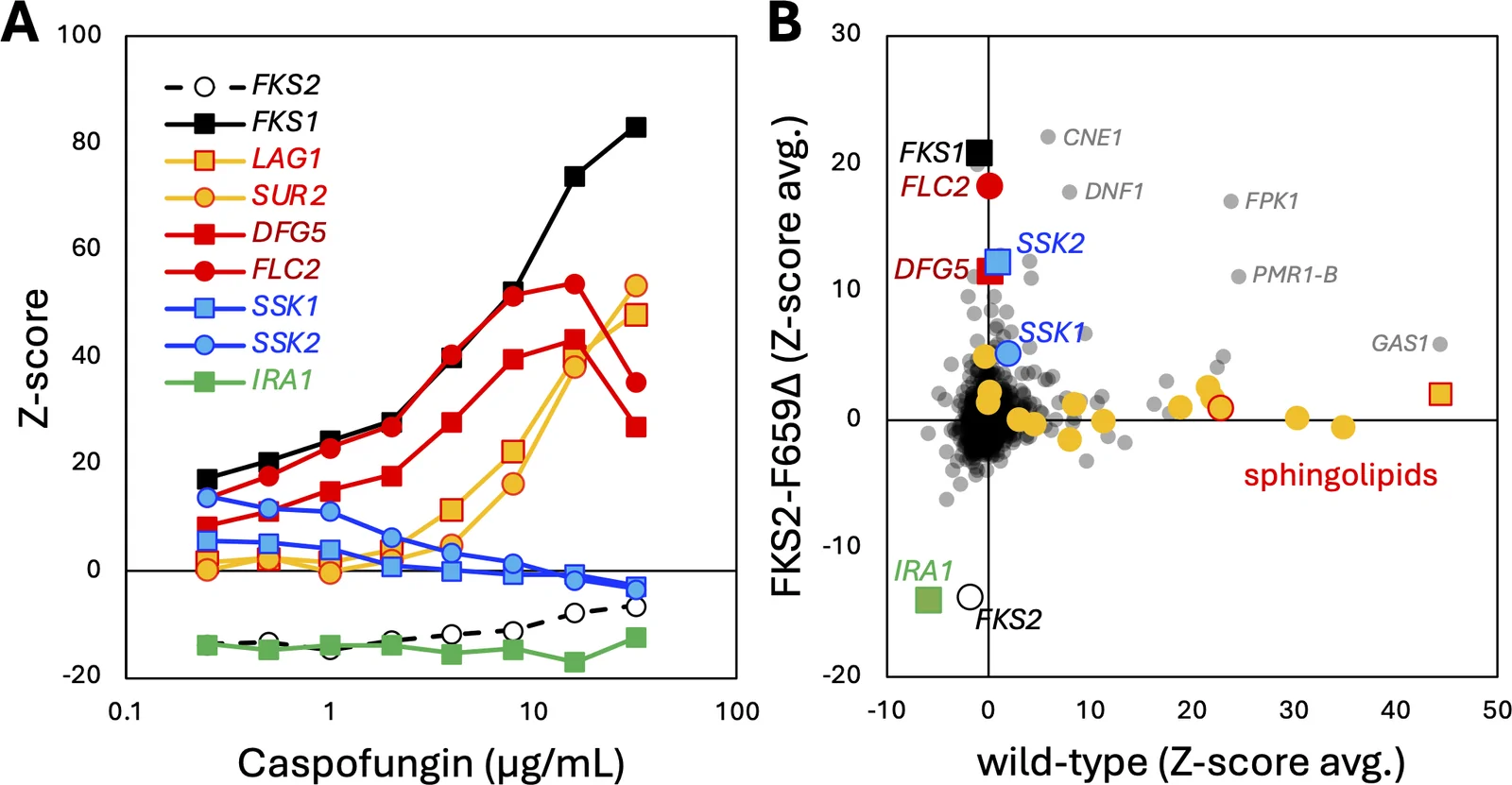
Tn-seq analysis of a FKS2-F659∆ mutant distinguishes regulators of heteroresistance and resistance**.** A diverse pool of *Hermes* transposon insertion mutants was generated in strain *FKS2-F659∆*, divided, diluted into fresh SCD medium containing the indicated doses of caspofungin, incubated for 24 h, and then analyzed by Tn-seq (see Materials and Methods) to identify genes with significant enrichment or depletion relative to the drug-free control cultures (positive or negative *Z*-scores). (**A**) *Z*-scores of selected genes were charted at each dose of caspofungin. (**B**) *Z*-scores of all genes in strain *FKS2-F659∆* at the three lowest doses of caspofungin were averaged and charted against average *Z*-scores obtained previously from wild-type parent strain BG14 exposed to caspofungin (30). Genes involved in sphingolipid biosynthesis (orange symbols) and selected other genes (colored symbols) are shown.

Tn-seq quantifies the growth of many different transposon insertion mutants simultaneously during exposure to varying doses of antifungals and therefore may serve as a high-throughput PAP assay. To test this idea, a diverse pool of Hermes-NAT1 transposon insertions was generated in the *FKS2-F659∆* mutant background, cultured in medium containing a broad range of caspofungin doses (0.25 to 32 µg/mL), and analyzed by Tn-seq (30). Caspofungin was utilized in this experiment rather than micafungin to avoid the large enrichment of sequence reads in mitochondrial genes that confer micafungin resistance when disrupted (30). The raw data were tabulated gene-wise, and *Z*-scores for each gene were calculated in every condition as before (30). *Z*-scores above 2.57 or below −2.57 correspond to *P*-values less than 0.01 and indicate gene disruptions that become significantly over- or under-represented relative to the average behavior of genes of similar size. As expected, transposon insertions in *FKS2* produced strongly negative *Z*-scores (ranging from −14.7 to −6.5) at all eight doses of caspofungin due to the complete loss of resistance and heteroresistance conferred by the *FKS2-F659∆* mutation (Fig. 5A). The *CRZ1* gene also exhibited mild under-representation at all doses of caspofungin (average *Z* = −2.0). *RLM1* was negative but not significant (average *Z* = −0.4), though *SMP1* (a paralog deriving from an ancient whole-genome duplication event) was significantly underrepresented (average *Z* = −3.0). *SMP1* has not been studied in *C. glabrata*, but functional redundancy between Rlm1 and Smp1 might explain the mild loss of heteroresistance observed in the *FKS2-F659Δ rlm1Δ* double mutant (Fig. 3C). As expected, transposon disruption of the *FKS1* gene produced extremely high *Z*-scores at all doses of micafungin (Fig. 5A, black squares), probably due to the increased expression and function of *FKS2-F659∆*.

Earlier Tn-seq experiments using the wild-type parent (strain BG14) identified 12 genes required for sphingolipid biosynthesis that could confer resistance to caspofungin when disrupted with transposons (30). Seven of those genes, plus two additional ones involved in sphingolipid biosynthesis (*AUR1* and *SCS7*), exhibited very positive *Z*-scores in the *FKS2-F659∆* mutant background, but the strongest effects were restricted to the four highest doses of caspofungin (4 to 32 µg/mL; Table S1). Two representative sphingolipid genes (*LAG1* and *SUR2*) were charted as a function of caspofungin dosage (Fig. 5A, orange symbols). Together, these findings show that *FKS2-F659∆* was necessary for resistance at all doses of caspofungin and that sphingolipids were still required for inhibition of Fks2-F659∆ enzymes at the highest doses.

At the lowest doses of caspofungin, the Fks2-F659∆ enzyme was probably insensitive to caspofungin due to its much weaker affinity for the drug (14). Nevertheless, *FKS1* and dozens of other genes exhibited positive *Z*-scores at low doses, indicating their disruption with transposons still improved fitness in these conditions, possibly due to increased expression of *FKS2-F659∆* or increased function of the encoded enzyme, Fks2-F659∆. To help distinguish resistance from heteroresistance effects, the *Z*-scores at three lowest doses of caspofungin were averaged for each gene in this *FKS2-F659∆* mutant data set and then charted against the average *Z*-scores from the wild-type parent strain obtained previously (Fig. 5B). The correlation was poor (*R*^2^ = 0.051), and the sphingolipid genes clearly stood out as being more important for resistance of the wild-type parent strain than heteroresistance of the *FKS2-F659∆* mutant strain (orange symbols) at these doses of caspofungin. Conversely, *FKS1* and several other genes stood out with highly positive *Z*-scores only in the *FKS2-F659∆* mutant. Two genes that regulate the HOG signaling pathway (*SSK1* and *SSK2*; blue symbols) (45) exhibited positive *Z*-scores in *FKS2-F659∆* at the lowest doses of caspofungin and then decreased at higher doses (Fig. 5A). *FKS1* exhibited strongly positive *Z*-scores at low doses and even higher *Z*-scores at the highest doses (black symbols). Two genes (*DFG5* and *FLC2*; red symbols) exhibited patterns similar to *FKS1*. The products of these two genes function together in a pathway or complex that attaches glycosylphosphatidylinositol (GPI)-anchored proteins in the plasma membrane to the beta-1,3,-glucan in the cell wall that is synthesized by Fks2 and Fks1 (40). The consistency observed for co-functional genes highlights the reproducibility of the Tn-seq method. The different patterns of *Z*-scores observed between the different sets of co-functional genes suggest complexity in the regulation of resistance and heteroresistance.

**Fig 6:**
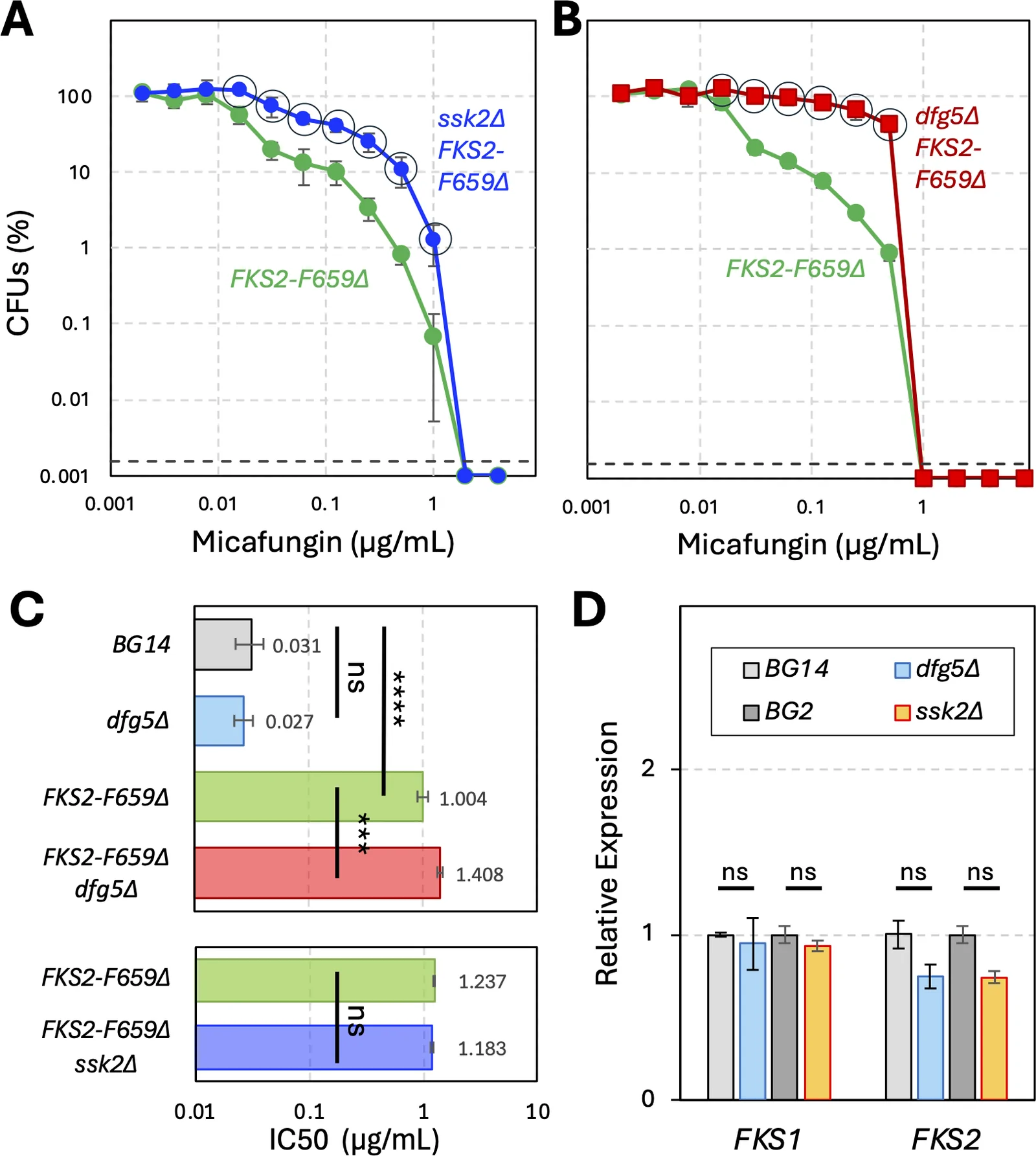
*SSK2* and *DFG5* regulate heteroresistance through post-transcriptional effects. (**A and B**) PAP assays comparing *ssk2∆ FKS2-F659∆* and *dfg5∆ FKS2-F659∆* double mutants to the *FKS2-F659∆* control strain were performed as described in Fig. 1. Open circles indicate double mutant samples where CFUs differed significantly from the control (*P* < 0.05; Welch’s *t*-test). (**C**) Broth microdilution assays of the indicated strains were performed in medium containing micafungin, and IC50 (average ± SD) was shown. ***P < 0.005, ****P < 0.001; Welch’s *t*-test). (**D**) qRT-PCR analysis of *FKS1* and *FKS2* gene expression in the indicated strains (average of 4 replicates ± SD). ns (*P* > 0.05)

We chose *SSK2* and *DFG5* for further analysis because the first gene is naturally disrupted in some strains of *C. glabrata* (30, 45), and the second encodes an enzyme that closely associates with *FKS2* in terms of expression pattern and biological function (40). When introduced into the heteroresistant *FKS2-F659∆* strain background, the *ssk2∆* mutation caused CFU to increase by 5- to 10-fold over the entire transitional range (Fig. 6A) but did not alter micafungin resistance (Fig. 6C). Even so, survivorship of the *ssk2∆ FKS2-F659∆* double mutant did not reach 100% like that of the *fks1∆ FKS2-F659∆* double mutant. Thus, the MAPKKK enzyme encoded by *SSK2* may antagonize heteroresistance associated with *FKS2-GOF* in strains of *C. glabrata* where it is functional. In contrast, a *dfg5∆ FKS2-F659∆* double mutant exhibited near 100% viability over most of the transitional range of micafungin as compared to the *FKS2-F659∆* parent strain (Fig. 6B), resembling *fks1∆ FKS2-F659∆* mutant cells. The *dfg5∆* mutation slightly increased resistance to micafungin in the *FKS2-F659∆* background but not the wild-type background (Fig. 6C). However, neither the *ssk2∆* nor *dfg5∆* mutants exhibited elevated expression of *FKS2* mRNAs (Fig. 6D). Thus, *SSK2* and *DFG5* seemed to antagonize *FKS2-F659∆* functions at post-transcriptional levels. As stress, calcineurin, and Crz1 signaling can induce expression of *DFG5* (40), its heterogeneous expression may cause heterogeneous antagonism of Fks1-F659∆ enzymes and contribute to echinocandin heteroresistance in clonal populations.

### *IRA1* increases heteroresistance and decreases resistance through differential effects on *FKS2* and *FKS1* expression

**Fig 7:**
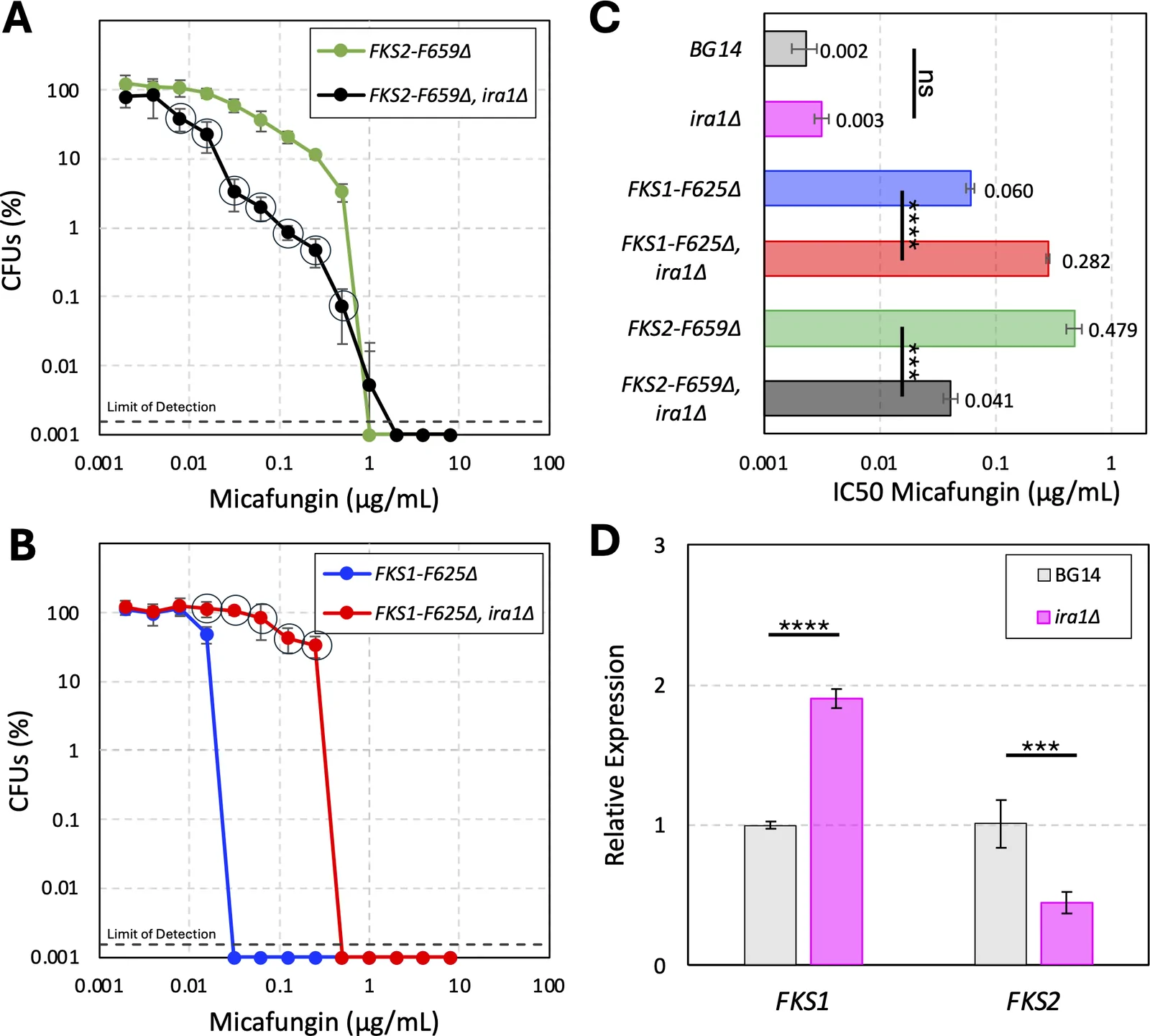
Ira1 regulates heteroresistance and resistance**.** The *IRA1* gene was knocked out in the *FKS1-F625∆, FKS2-F659∆*, and wild-type BG14 parent strain backgrounds, and the resulting strains were subjected to PAP assays using micafungin as described in Fig. 1 (**A and B**). Open circles indicate treated or double mutant samples where CFUs differed significantly from control (*P* < 0.05, Welch’s test). The strains were also subjected to broth microdilution assays to quantify micafungin resistance (**C**). Bars indicated average IC50 of four replicates (±SD). (**D**) qRT-PCR analysis of *FKS1* and *FKS2* gene expression was performed on wild-type, *ira1*∆, *fks1∆*, and *fks2∆* mutant derivatives of strain BG2 (average of 4 replicates ± SD). Statistical significance was assessed using a Welch’s *t*-test (ns *P* > 0.05, ***P < 0.005; ****P < 0.001).

Genes that increase *FKS2-F659∆* expression or function should exhibit negative *Z*-scores similar to those of *FKS2* across all tested doses of caspofungin. A total of 30 genes exhibited average *Z*-scores less than −2.57, corresponding to *P*-value < 0.01, across all doses of caspofungin in the *FKS2-F659∆* mutant (Table S1). Gene ontology (GO) term analysis (Table S2) (46) indicated this set was weakly enriched for components of the m-AAA complex (*YTA12* and *AFG3*) in the mitochondrial inner membrane that processes transit peptides. Further, two genes involved in chitin biosynthesis (*CHS3* and *CHS7*) were included in this set of 30 genes. By far, the strongest hit was *IRA1* (green symbols; Fig. 5A and B), which encodes an inhibitor of the small GTPase Ras1 that increases cAMP levels and signaling by protein kinase A in response to glucose availability in *S. cerevisiae* (29). To test whether *IRA1* promotes heteroresistance in *C. glabrata*, the gene was knocked out of *FKS2-F659∆* mutants and wild-type strains, and PAP assays were performed. CFUs in the transitional range of micafungin decreased 20-fold (averaged between 0.007813 and 0.5 µg/mL) in the *FKS2-F659∆ ira1∆* double mutant (TR = 8) relative to *FKS2-F659∆* (Fig. 7A), consistent with a role for Ira1 in positively regulating expression or function of *FKS2-F659∆*. We then tested whether *IRA1* exhibited effects on *FKS2-F625∆*. Surprisingly, the *FKS1-F625∆ ira1∆* double mutant exhibited strongly increased resistance to micafungin relative to the parent strain, as indicated in the PAP assay (Fig. 7B) as well as broth microdilution assay (Fig. 7C). The same patterns were observed in broth microdilution assays using caspofungin (Fig. S6). *IRA1* therefore served as a positive regulator of *FKS2-F659∆* and a negative regulator of *FKS1-F625∆*. Quantitative real-time PCR was utilized to test whether *IRA1* regulated expression of *FKS2* or *FKS1*. Consistent with the PAP assays, *FKS1* expression was nearly doubled, and *FKS2* expression was halved in the *ira1Δ* strain, relative to the parent strain (Fig. 7D). This indicates that Ira1 plays different roles in the expression of the *FKS* genes.

## DISCUSSION

**Fig 8:**
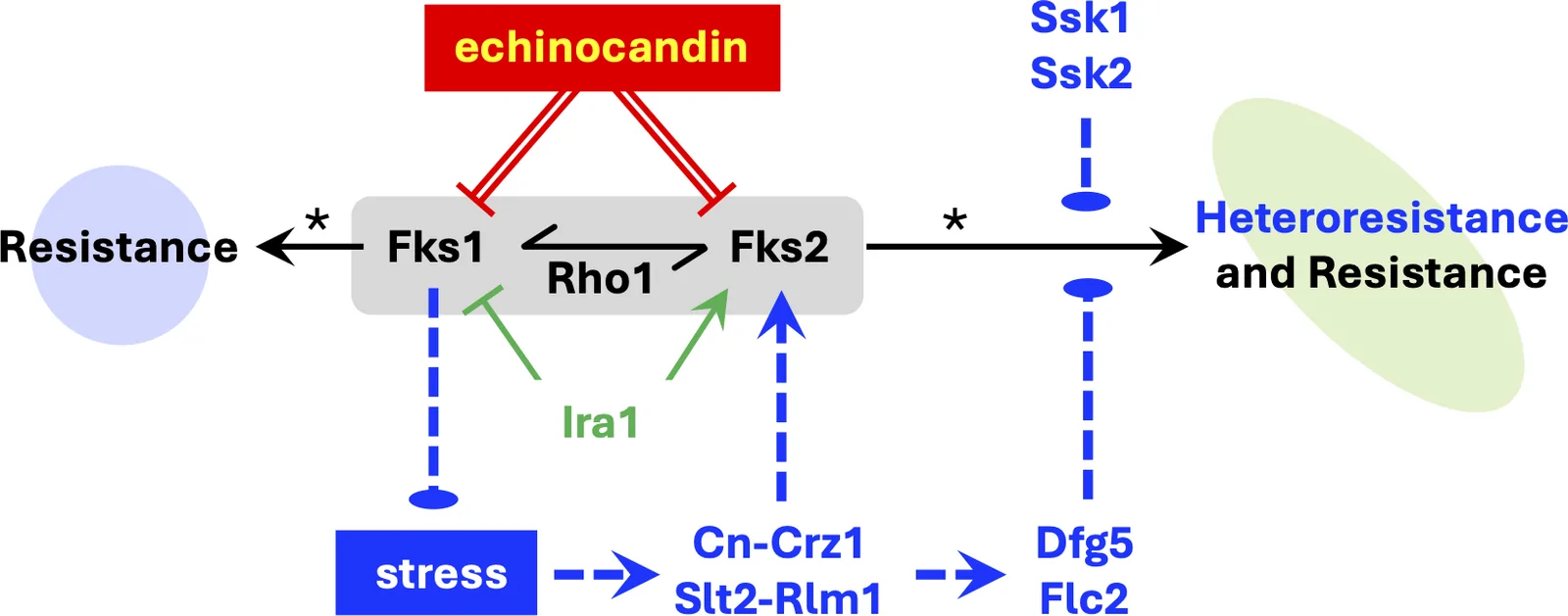
Working model of genes regulating echinocandin resistance and heteroresistance in *C. glabrata*. Echinocandins inhibit wild-type Fks1 and Fks2 enzymes. When one enzyme contains a GOR mutation in hotspot-1 (thus removing a red double arrow), the other remains inhibited at normal doses and antagonizes the function of resistant enzyme by sequestering Rho1 or by another mechanism (see Discussion). This mutual antagonism can be abolished by a secondary loss-of-function or gain-of-resistance mutation in the antagonizing gene (removing the remaining red double arrow and causing super-resistance). Heterogeneous expression of *FKS2-GOR* and heterogeneous regulation of its function in clonal populations can contribute to echinocandin heteroresistance. Potential sources of cell-to-cell heterogeneity include stress variation, signaling variation via stress-sensing pathways such as calcineurin-Crz1 and Slt2-Rlm1, and Ssk2-Hog1 leading to variable expression or function of post-transcriptional regulators such as Dfg5-Flc2 (dashed blue lines). Ira1 decreased expression of *FKS1* and increased expression of *FKS2,* thereby enhancing resistance and heteroresistance of the GOR strains, respectively (green arrows).

This study demonstrates that GOR mutations in *FKS2* associated with therapeutic failure in patients can also produce high levels of progressive heteroresistance to echinocandins *in vitro*. Deeper investigations into the genetic regulation of heteroresistance suggest, but do not yet prove, that cell-to-cell variation in both expression of the *FKS2-GOR* gene and regulation of the Fks2-GOR gene product may combine to generate a diverse population of *C. glabrata* cells where a significant fraction can survive the initial plating on high doses of echinocandins and produce enough viable daughter cells to form a colony on agar medium (summarized in Fig. 8). In particular, the stress-sensing calcineurin-Crz1 and Slt2-Rlm1 signaling pathways known to upregulate *FKS2* gene expression were each required for maximal CFU formation in the transitional range of echinocandin doses. A recent study showed broad cell-to-cell variation in the expression of functional halo-tagged Fks2 protein in unstressed populations of *C. glabrata* cells (10). Brief exposure of unstressed *FKS2-GOR* mutants of *C. glabrata* to a calcineurin inhibitor (FK506) significantly decreased the ability to form colonies within the transitional range of echinocandins. Conversely, brief pre-exposure to an ER stressor (manogepix), which activates calcineurin signaling (40), increased CFUs in the transitional range of micafungin. These findings suggest colony formation is sensitive to the status of the plated cells, in addition to stress signaling pathways that may be necessary for survival of the daughter cells. Interestingly, a genome-wide Tn-seq screen revealed several negative regulators of heteroresistance (e.g., *SSK2* and *DFG5*) that appeared to act on *FKS2-GOR* function at post-transcriptional levels. *SSK2* encodes a MAPKKK that regulates Hog1 signaling (45). *DFG5*, whose expression is also controlled by calcineurin-Crz1 (40), encodes an enzyme at the cell surface that attaches GPI-anchored proteins to beta-1,3-glucan synthesized by Fks2 (47). Precisely how these proteins negatively regulate the heteroresistance of *FKS2-GOR* mutants remains to be elucidated. Nevertheless, disabling mutations in *SSK2* have arisen in diverse strains of *C. glabrata* (45), potentially impacting echinocandin heteroresistance as well as resistance (30).

In contrast to the strong heteroresistance observed for *FKS2-GOR* mutants, the identical GOR mutations in *FKS1* produced little or no heteroresistance despite conferring strong resistance to echinocandins. Expression of *FKS1* appeared unresponsive to the stress signaling pathways and more constitutive, possibly producing less variation of Fks1 protein levels from cell to cell. Flow cytometry of cells expressing halo-tagged Fks1 could be used to test that prediction (10). Resistance of *FKS1-F625∆* mutants to echinocandins was not strongly affected by calcineurin inhibitors either in broth microdilution assays (8) or PAP assays, suggesting that calcineurin signaling did not strongly impact its expression or function. However, the *FKS1-F625∆* mutants of *C. glabrata* analyzed here did indeed exhibit CFUs over a narrow transitional range of micafungin doses, suggesting the acquisition of mild heteroresistance. Loss of the *IRA1* gene, which encodes a negative regulator of the Ras1-cAMP-PKA signaling pathway in *S. cerevisiae* (29), strongly increased expression of *FKS1* and resistance of *FKS1-F625∆* to micafungin but did not expand the transitional range in PAP assays. This mild heteroresistance conferred by the Fks1-F625∆ protein may be due, at least partly, to some additional heterogeneity in expression or regulation by the drug-bound Fks2 protein.

Resistance observed in *FKS1-GOR* mutants and *FKS2-GOR* mutants has long been known to be antagonized by the presence of the echinocandin-sensitive paralog (8). Consequently, “super-resistance” can arise through secondary mutations where the paralog was either destroyed (8, 48) or mutated with a secondary GOR mutation (17, 49). Such super-resistance has also been observed in clinical isolates from patients receiving echinocandin therapies (8). Based on previous studies in *S. cerevisiae*, at least three different, but non-exclusive, hypotheses could explain the observed antagonism between drug-bound Fks proteins and drug-free Fks-GOR proteins. First, drug-bound Fks proteins could sequester Rho1 and thereby lower the activity of the Fks-GOR proteins, particularly if Rho1-GTP levels are limiting, which seems to be the case in *S. cerevisiae* (50). Second, drug-bound Fks proteins could directly sequester the drug-free Fks-GOR proteins into heterodimers with reduced activity. Consistent with this idea, inactive homodimers of Fks1 that are bridged by a tRNA molecule and occluded from Rho1 binding have been isolated recently from *S. cerevisiae* and visualized by cryo-electron microscopy (51). Third, drug-bound Fks proteins may occupy important sites in the cell membrane and prevent access of those sites to the active drug-free Fks-GOR enzymes. Halo-tagged Fks1 and Fks2 proteins appeared to occupy partially overlapping but still distinct domains in the growing buds of otherwise wild-type *C. glabrata* (10). In any of these scenarios, variable expression of *FKS2* in the population could produce heterogeneous levels of antagonism in *FKS1-GOR* mutants and thereby increase heteroresistance. These hypotheses surrounding antagonism are not mutually exclusive, and each may contribute significantly to the observed patterns of resistance and heteroresistance in *C. glabrata*.

The mechanisms proposed here may help explain echinocandin heteroresistance in other *Candida* species. In *C. parapsilosis*, where echinocandin heteroresistance has been observed in many isolates and linked to therapeutic failure (25), a natural gain-of-resistance mutation already exists in hotspot-1 of *FKS1* (31). Heterogeneous expression of this natural *FKS1-GOR* gene, or variable levels of antagonism by either Dfg5 or the drug-bound Fks2 or Fks3 proteins could contribute to the progressive heteroresistance observed in some of the clinical isolates (25). The *FKS2* and *FKS3* genes are highly expressed in *C. parapsilosis* (31), and FK506 blocks *C. parapsilosis* growth in laboratory conditions (52), suggesting the species may experience stresses in laboratory environments. On the other hand, some isolates of *C. parapsilosis* exhibited non-progressive heteroresistance, which could arise from unstable aneuploidy, unstable copy number variation, or other two-state mechanisms that remain to be discovered. In *C. albicans*, the non-essential *FKS2* and *FKS3* gene products may antagonize the essential *FKS1* gene product that is susceptible to echinocandins (53). When gain-of-resistance mutations in *FKS1* arise heterozygously in diploid *C. albicans*, the resistance is initially antagonized by the drug-sensitive allele, and super-resistance occurs upon secondary loss-of-function mutations or gain-of-resistance mutations in that allele (54). Echinocandin heteroresistance in *C. albicans*, which has not yet been reported (24), may only arise when gain-of-resistance mutations in *FKS1* become homozygous (diminishing antagonism by the wild-type *FKS1* allele) and when antagonism by *FKS2* and *FKS3* becomes elevated heterozygously in the population. An instance of echinocandin heteroresistance reported in *C. auris* (25) also may be explained by any of these mechanisms.

The mechanisms of echinocandin heteroresistance in *C. glabrata* may be distinct from mechanisms of echinocandin tolerance and persistence, which are starting to be elucidated (19). Tolerance and persistence mechanisms increase the lifespan of the major and minor subpopulations, respectively, of cells exposed to extremely high doses of echinocandins that are far above the MIC (55). Though brief stress (e.g., manogepix pretreatment) and calcineurin signaling strongly increased tolerance of wild-type *C. glabrata* to echinocandins, the effects were largely independent of Crz1 and completely independent of *FKS2* (19). Calcineurin therefore sits at a critical control point in *C. glabrata* that confers tolerance and persistence to echinocandins in wild-type strains in addition to resistance and heteroresistance in strains bearing *FKS2-GOR* mutations. The findings presented here suggest that short-term administration of tacrolimus (FK506) or other inhibitors of fungal calcineurin could improve clinical outcomes by blocking all these undesirable phenomena associated with antifungal therapies, while potentially avoiding side effects (immunosuppression, nephrotoxicity, etc.) that arise from long-term use.

Heteroresistance to azole-class antifungals has also been detected in a broad survey of *C. glabrata* clinical isolates (18) and in other fungal species (20, 21, 56, 57). Progressive heteroresistance to fluconazole in many *C. glabrata* clinical isolates may be linked to heterogeneous expression of *CDR1* (18), which encodes a powerful ATPase that effluxes the antifungal. Heterogeneous expression of *ERG11*, encoding the target of azole-class antifungals that is also regulated by calcineurin signaling in *C. glabrata* (58), represents another possible source of cell-to-cell variation. Non-progressive heteroresistance to azoles observed in *Cryptococcus neoformans* may be conferred by infrequent aneuploidy that results in overexpression of the target (*ERG11*) and an efflux pump (20). Tolerance to azole-class antifungals has long been known to depend on calcineurin signaling in *C. glabrata*, *C. albicans*, and several other fungal species (43, 59, 60). Deeper exploration of how calcineurin signaling achieves all these anti-antifungal effects could spur the development of non-immunosuppressive compounds that augment the efficacy of multiple antifungal classes.

Standard clinical tests of antifungal susceptibility often fail to detect heteroresistance, contributing to the use of ineffective treatment regimens (26–28). While improved diagnostic methods must be developed to meet this challenge, the molecular mechanisms that drive all types of heteroresistance should be elucidated experimentally. As shown here, unbiased Tn-seq methods have the power to screen large numbers of mutants for effects on heteroresistance in addition to resistance. While Tn-seq can produce novel insights, PAP assays remain the gold standard for mechanistic studies on engineered strains. Earlier approaches have utilized only one or two parameters to summarize PAP assay data, such as the width of the transitional range, the average viability (CFU) across the transitional range, or the area under the combined curve. Because such summaries cannot distinguish progressive and non-progressive forms of heteroresistance, additional parameters or more nuanced modeling approaches will be required for mechanistic studies and potentially lead to improvements in clinical diagnostics.

## MATERIALS AND METHODS

### *C. glabrata* strains and plasmids

All strains and their sources are listed in Table S3. *C. glabrata* strains were derived from BG14, a *ura3∆* derivative of wild-type strain BG2 (61). Individual gene knockouts were constructed using the PRODIGE method (62), where coding sequences were replaced with the coding sequence of *URA3 or HIS3* from *S. cerevisiae* plasmids pRS406 or pRS403, respectively (63). Colony PCR was used for screening validation. For this study, mutant *FKS1* and *FKS2* genes were PCR-amplified from *FKS1-F625∆* and *FKS2-F659∆* in 2001-HTU (8) (received from David Perlin) and transformed into strain BG14. All oligonucleotides used for generating and screening mutants are available in Table S4. pCU-pFKS1-FKS2-F659Δ was generated using custom plasmid synthesis (GenScript). The plasmid carried 1,000 bp upstream of the *FKS1* start codon and the coding sequence of *FKS2-F569Δ* cloned into the pCU-MET3 vector using the SacI and XhoI restriction sites.

### Antifungals and key chemicals

Micafungin (Cat. #18009) was obtained from Cayman Chemicals and caspofungin (Cat. #S3073), tacrolimus (FK506) (Cat. #S5003), and manogepix (E1210) (Cat. #S0491) from SelleckChem. 5-Fluoroorotic acid (5-FOA) was obtained from Zymo Research (Cat. # F9001-5), and ClonNAT (Nourseothricin Sulfate) was obtained from GoldBio (Cat. #N-500-100).

### Tn-seq screen for genes that regulate caspofungin heteroresistance

A new pool of Hermes-NAT1 insertion mutants in *FKS2-F659∆ ura3∆* strain was generated by transforming the strain TJN142 with pCU-MET3-Hermes and then inducing transposition in 40 replicate cultures, as described previously (64). Replicate cultures were pooled and enriched in 5-FOA and NAT and then cryo-preserved as previously described (64). The pool of insertion mutants was thawed from storage at −80°C, grown to saturation, then diluted into fresh synthetic complete dextrose (SCD)-0 medium containing a range of caspofungin doses, and shaken at 30°C for 48 h (30). No replicate samples or pools were prepared or analyzed. The cells were pelleted and washed, and then genomic DNA (gDNA) was extracted using the Quick-DNA Fungal/Bacterial Miniprep Kit from Zymo Research (Cat. #D6005). gDNA was fragmented by sonication, repaired, ligated to adapters, and size-selected, and then insertion sites were PCR amplified as described previously (65). Single-end reads were sequenced using AVITI 2 × 75 Sequencing Kit Cloudbreak FS High Output (Cat. # 860-00015) with custom primers specific to the HERMES transposon and the splinkerette to ensure that all reads were from genomic DNA at the transposon insertion site (65).

Samples were basecalled and demultiplexed using Bases2Fastq software (v2.1.0) by Element Biosciences, and CutAdapt was used to identify and trim away adapter sequences in the case of adapter readthrough (66) and aligned to the BG2v1 reference genome (67) using Bowtie2 (68). Mapped reads with low quality (*Q* < 20) or mismatches at position 1 were discarded, and the remainder were tabulated for each site in the genome. The total number of sequence reads in each annotated gene was tabulated, and the top site was removed to limit passenger effects (69). *Z*-scores were calculated for each gene at every caspofungin dose relative to untreated sample exactly as before (30) (Table S1). Briefly, the log-2 fold-change of normalized read counts for test over control was divided by the local standard deviation, which was computed for each gene based on the averaged read counts for test and control as the input variable in a power function with best-fit parameters that were obtained from a comparison of two replicate control samples from the same sequencing run (30). All *Z*-scores are listed in Table S1, together with previously published *Z*-scores obtained in strain BG14 and averages of *Z*-scores from several dose ranges (charted in Fig. 5B).

### Population analysis profile (PAP) assays and analyses

Single colonies were grown to saturation overnight in SCD-0 media, shaking at 30°C. Solid yeast extract peptone dextrose (YPD) media containing varying doses of drug were prepared by adding drug to warmed, liquid media, and allowing plates to solidify at room temperature for at least 24 h. The saturated cultures were diluted 1:50 into fresh SCD-0 media and then serially diluted 1:5. Two microliters of each dilution was spotted onto agar YPD media containing varying doses of micafungin or caspofungin. Plates were incubated for 24 h at 30°C, and single colonies were counted manually using a dissecting microscope. All strains were tested in biological quadruplicate. Colony-forming units per mL and the percentage of CFUs for each replicate were calculated with respect to the no-drug control (unless otherwise indicated in the figure legend) at every drug concentration, and the arithmetic means and standard deviations were calculated at each drug concentration.

### Broth microdilution assays and analyses

Single colonies were grown to saturation overnight in SCD medium, shaking at 30°C. Cells were back-diluted 1:2,000 in fresh SCD medium with 1:2 serial dilutions of the antifungal in flat-bottom 96-well plates. Plasmid-bearing cells were grown and treated in SCD-U medium. Treated cells were grown 20–48 h without shaking at 30°C, shaken to resuspend, and OD600 was measured using an Accuris Instruments SmartReader 96T. All strains were tested in biological quadruplicate. The raw data were fitted to a sigmoid equation *y* = m1 + (m2 − m1) / (1 + (*x* / m3)^m4^ in Kaleidagraph (v5.04, Synergy), and the dose at which half-maximal growth was observed (IC50) was calculated independently for each replicate. The arithmetic mean and standard deviation were calculated for each strain.

### RT-qPCR experiments

Single colonies were picked and grown overnight, shaking at 30°C to saturation. For all samples, cells were diluted 1:50 in fresh SCD medium and later harvested by centrifugation (14 k, 60 s). The supernatant was aspirated, and cell pellets were flash-frozen in liquid nitrogen and stored at −80°C until RNA extraction. Total RNA was extracted from cells using a hot acid phenol-chloroform extraction protocol (70). Cells were lysed in RNA lysis buffer (6 mM NaOAc, 8.4 mM EDTA, 1% SDS), and RNA was purified through two phenol and one chloroform extraction. RNA was precipitated with isopropanol, resuspended in TE buffer, and treated with DNase (New England Biolabs). One microgram RNA was reverse transcribed using the High-Capacity cDNA Reverse Transcription Kit (Thermo). Real-time PCR was performed using the CFX96 Touch Real-Time PCR Detection System (Bio-Rad) using the ABsolute Blue QPCR Mix SYBR Green Kit (Thermo Fisher) with the following parameters: 15 min at 95°C, 40× (15 min at 95°C, 30 min at 58°C, 30 min at 72°C). Target gene transcript levels were normalized to averaged *TEF1* transcript levels in each sample, and this ratio from each sample was normalized to that of BG14 cells. Target primers are identified in Table S4.

## Data Availability

Raw sequencing reads used in this study were deposited at the NCBI Sequence Read Archive (SRA) with the BioProject ID PRJNA1454099. Caspofungin resistance data (30) were obtained from SRA BioProject ID PRJNA1247003. A tabulation of the chromosomal coordinates and frequency of each mapped transposon insertion site (site count files) and a tabulation of the number of mapped transposon sites that fall within annotated gene boundaries (gene count files) are included Tables S5 and S6.

## SUPPLEMENTAL MATERIAL

Supplemental figures aac.00549-26-s0001.pdf Fig. S1 to S6.

Table S1 aac.00549-26-s0002.xlsx *Z*-scores for all genes.

Table S2 aac.00549-26-s0003.xlsx GO term analysis.

Table S3 aac.00549-26-s0004.xlsx Strain list.

Table S4 aac.00549-26-s0005.xlsx List of oligonucleotides.

Table S5 aac.00549-26-s0006.csv Tabulation of sequence reads per site.

Table S6 aac.00549-26-s0007.xlsx Tabulated reads per gene.

ASM does not own the copyrights to Supplemental Material that may be linked to, or accessed through, an article. The authors have granted ASM a non-exclusive, world-wide license to publish the Supplemental Material files. Please contact the corresponding author directly for reuse.
