# Supplementary material for: A gene network regulating echinocandin heteroresistance in Candida glabrata
Source: Antimicrob Agents Chemother. 2026 Oct 8;70(11):e00549-26. doi: 10.1128/aac.00549-26 (PMC13731172; doi:10.1128/aac.00549-26)
Supplement: Supplementary file 1 [file aac.00549-26-s0001.pdf]

Supp. Figure 1 Harrington et al

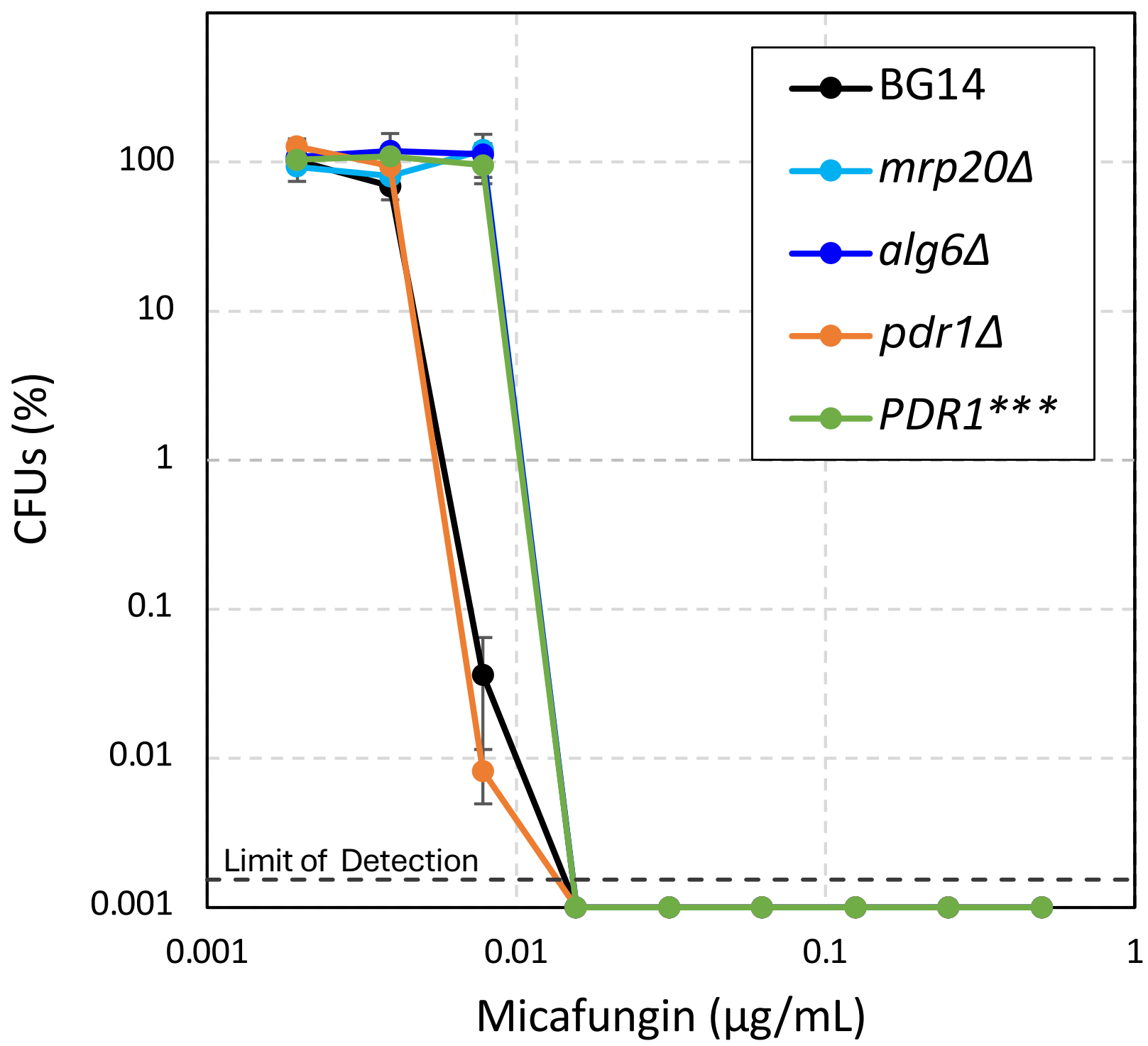

Figure S1. Mutations associated with pan-resistance are not heteroresistance. The indicated strains were subjected to PAP assays and CFUs were calculated as described in Figure 1. *PDR1**** refers to *PDR1-GOF*. Symbols indicate averages of four replicates (± SD). Limit of detection = 0.0015%, approximately 1 colony detected out of 4 biological replicates.

Supp. Figure 2 Harrington et al

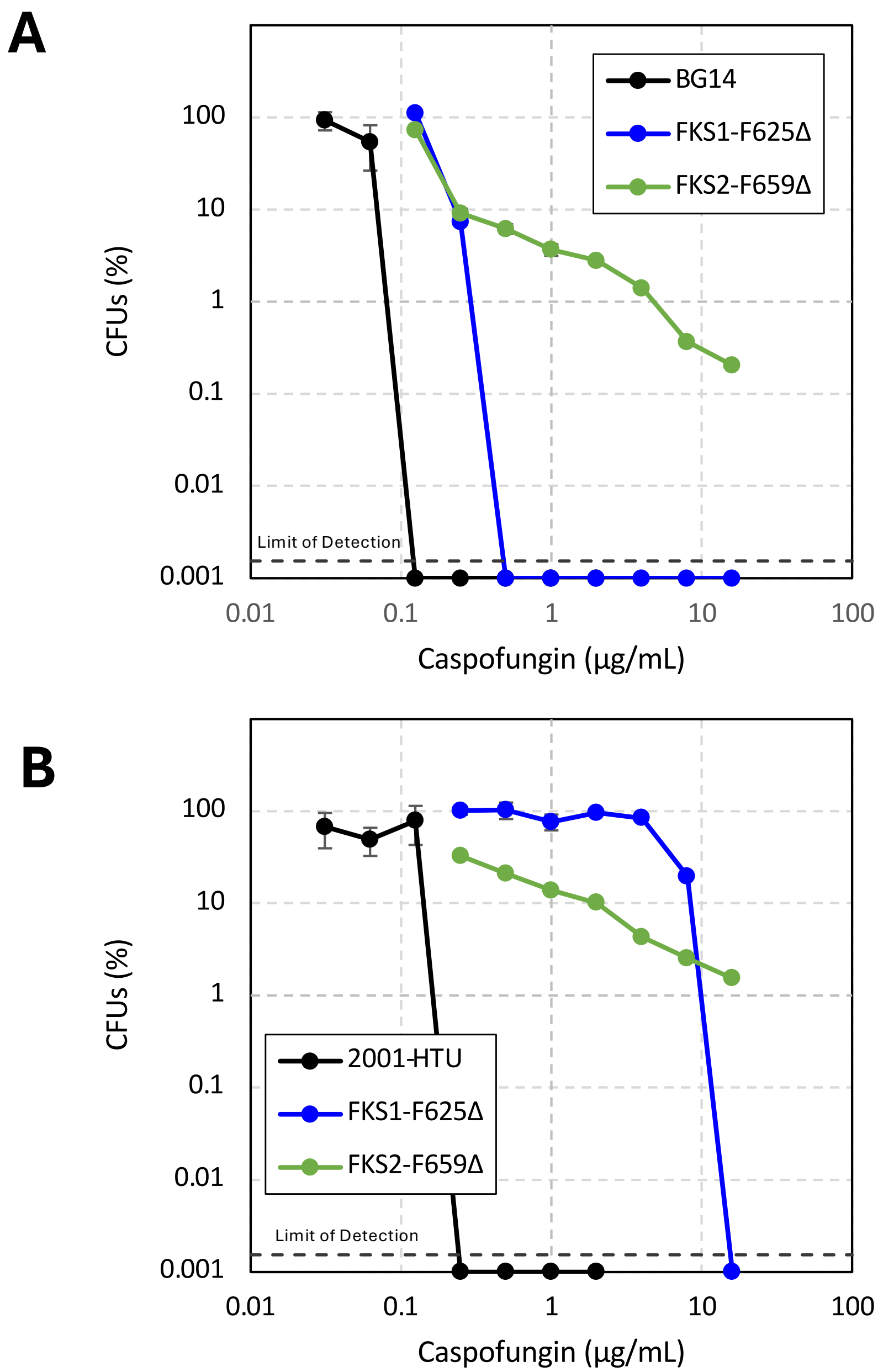

Figure S2. Caspofungin heteroresistance of *FKS2-F659Δ* strains of *C. glabrata*. PAP assays on the indicated strains were performed as described in Figure 1 except caspofungin was utilized instead of micafungin. Symbols indicate averages of four replicates (± SD). Limit of detection = 0.0015%.

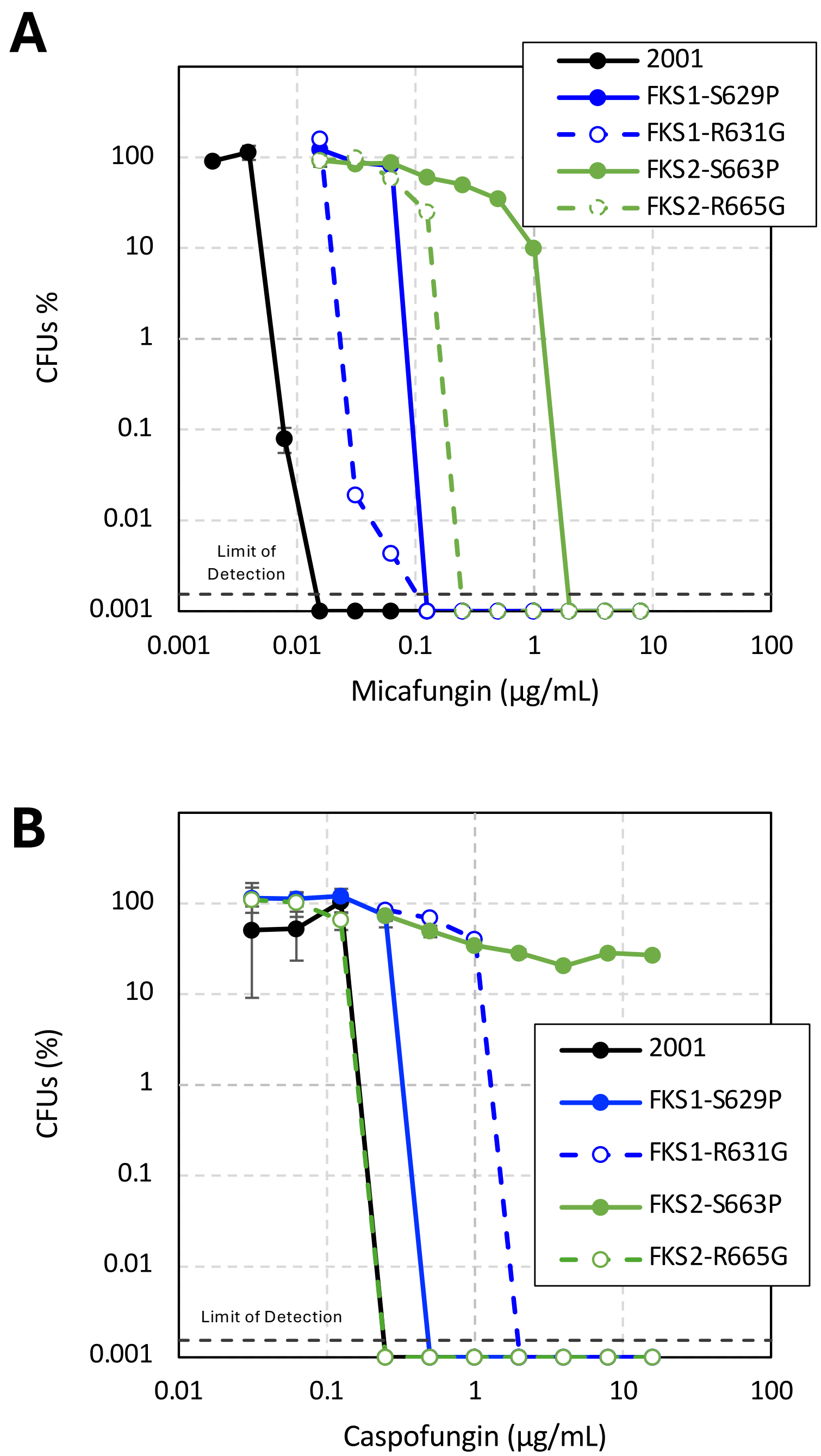

Figure S3. Hotspot 1 mutations in *FKS2*, not *FKS1*, exhibit heteroresistance to micafungin and caspofungin in the *C. glabrata* type strain background. (A-B) All strains were treated and CFUs were calculated as previously described in Figure 1. Symbols indicate averages of four replicates (± SD). Limit of detection = 0.0015%.

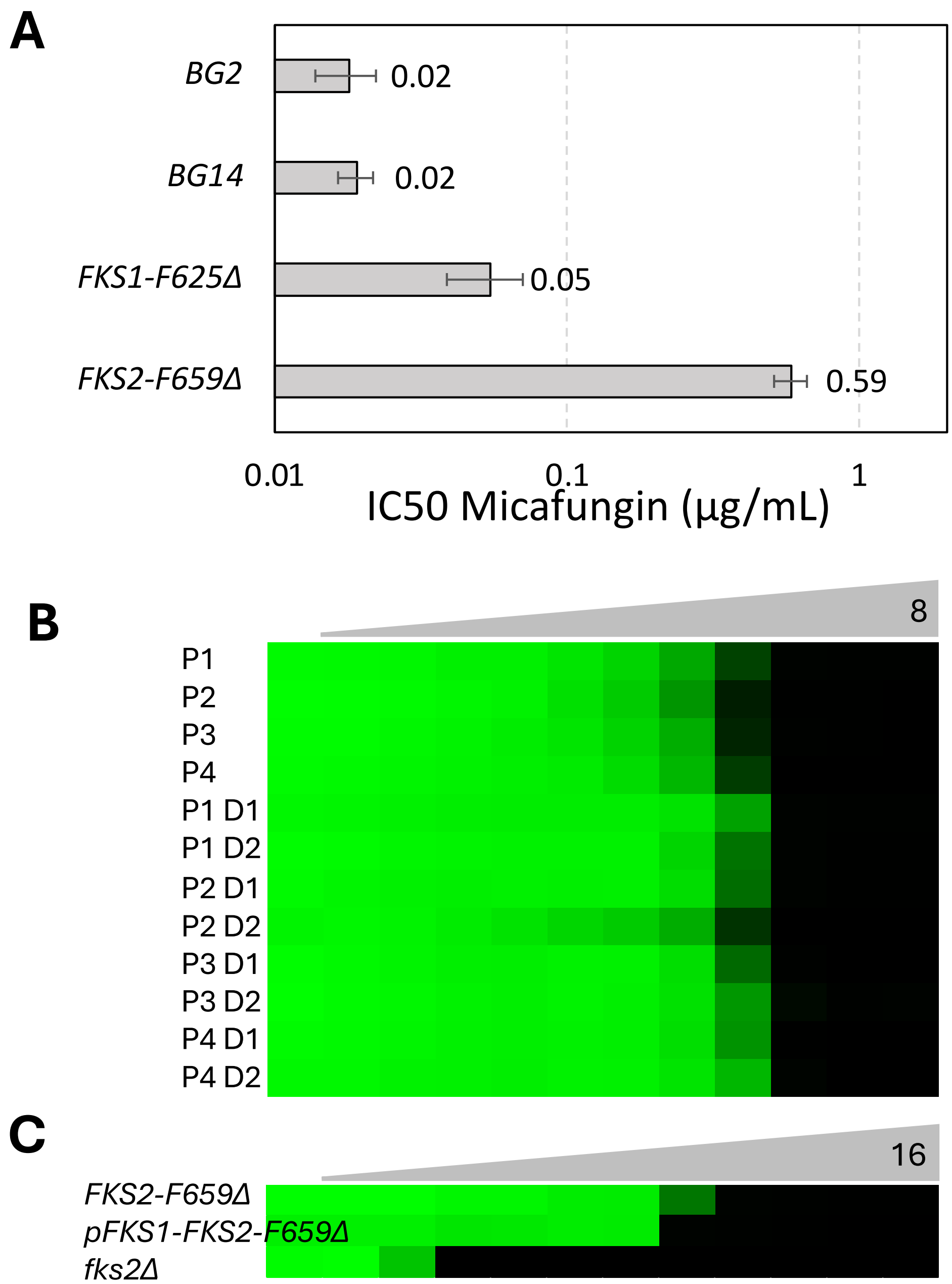

Figure S4. Broth microdilution assays of micafungin resistance. All strains were grown to saturation overnight in synthetic complete media and diluted 1:2000 in media containing two-fold dilutions of micafungin from a maximum of 8 µg/mL (A,B) or 16 µg/mL (C). OD600 was measured after 24 hours (A,B) or 48 hours (C) of incubation. (A) Four replicates of the indicated strains were individually fit to the sigmoid equation and the resulting IC50s were averaged (indicated right of bars) +/- SD. (B) Heat map of raw OD600 data from four replicates of the *FKS2-F659Δ* parent strain (P1 to P4) and two passaged descendants (D1, D2) of each parent. (C) Heat map of raw OD600 data averaged from four replicates of *FKS2-F659Δ* or *fks2Δ* mutants transformed with control plasmid pCU-MET3 or *fks2Δ* transformed with plasmid pCU-pFKS1-FKS2-F659Δ. Green = maximum OD600.

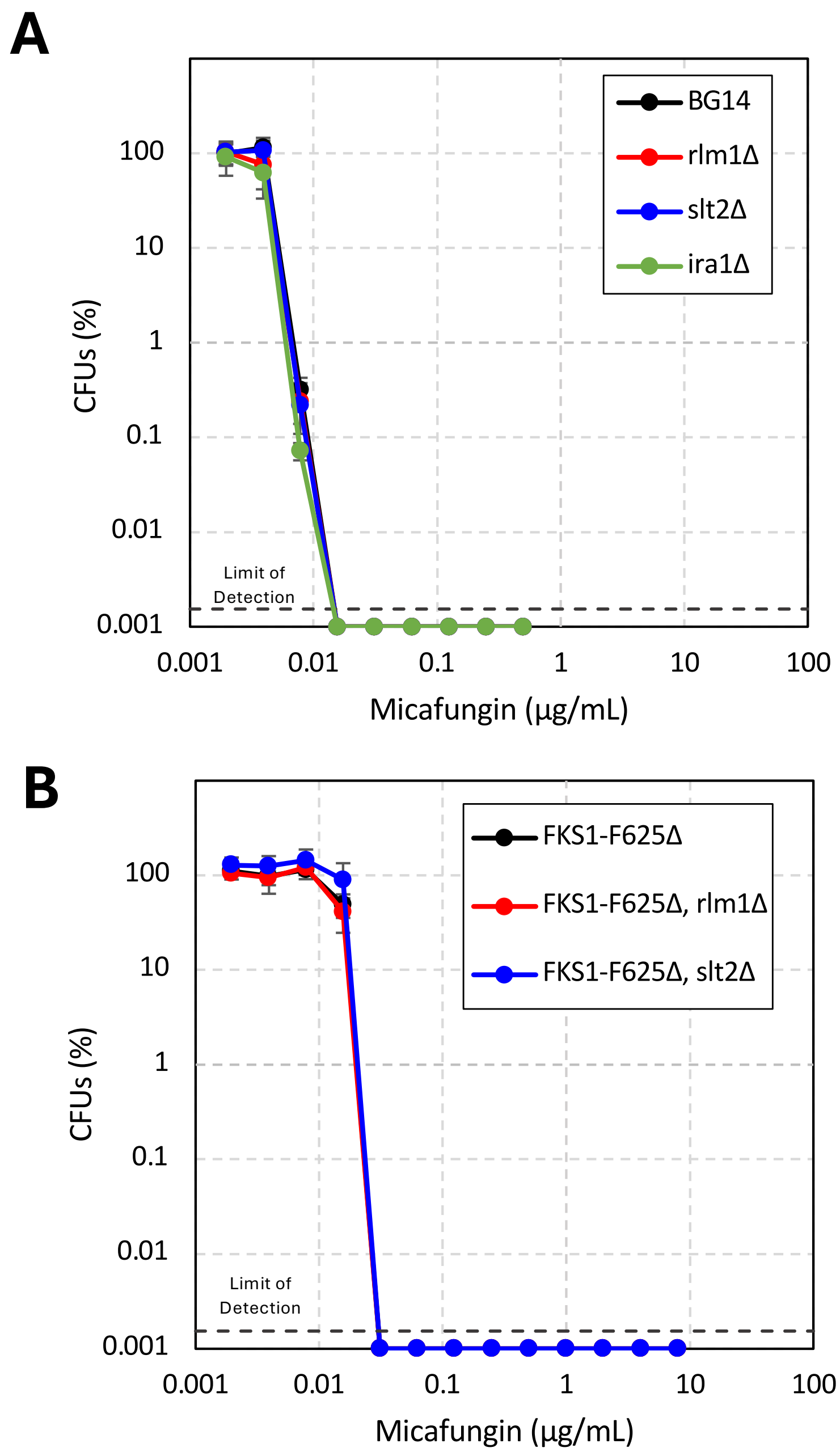

Figure S5. Positive regulators of *FKS2-F659Δ* heteroresistance have no effect in *FKS2* or *FKS1-F625Δ* strains of *C. glabrata*. PAP assays were performed on the indicated strains as described in Figure 1. Symbols indicate averages of four replicates (± SD). Limit of detection = 0.0015%.

Supp. Figure 6 Harrington et al

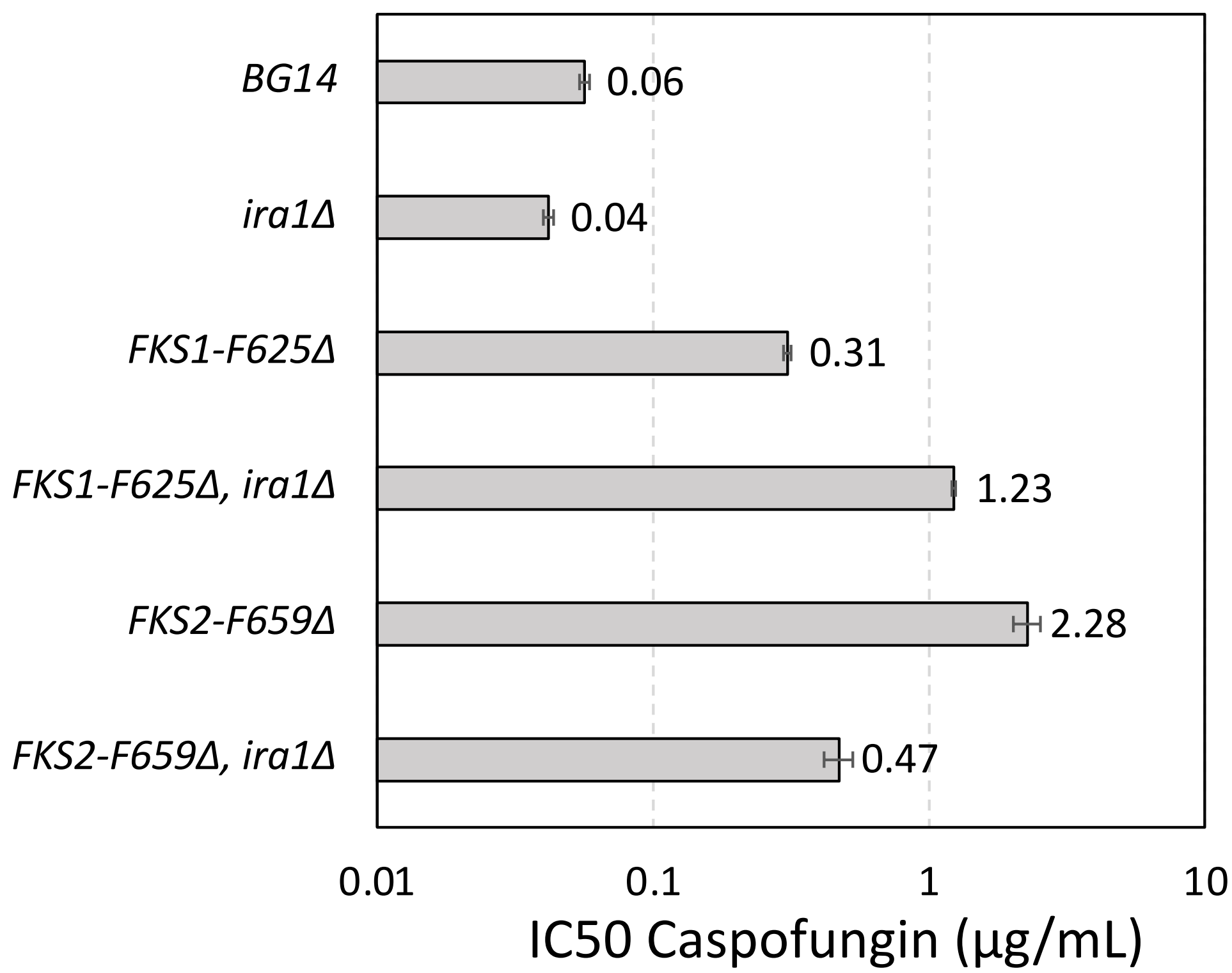

Figure S6. *IRA1* regulates caspofungin resistance in GOR strains of *C. glabrata*. All strains were grown to saturation overnight in synthetic complete media and diluted 1:2000 in media containing two-fold dilutions of micafungin. OD600 was measured after 24 hours of incubation, biological replicates (4) of each strain were fit to a sigmoid equation separately and the IC50 was averaged (indicated right of bars). Error bars represent +/- SD.
